# Macrophage incorporation into a 3D perfusion tri-culture model of human breast cancer

**DOI:** 10.1186/2051-1426-3-S2-P401

**Published:** 2015-11-04

**Authors:** Teresa M DesRochers, Lillia Holmes, Lauren O'Donnell, Christina Mattingly, Stephen Shuford, Mark A O'Rourke, Mary B Rippon, William J Edenfield, Matthew R Gevaert, David E Orr, Howland E Crosswell

**Affiliations:** 1 KIYATEC, Inc., Greenville, SC, USA; 2 Greenville Health System, Greenville, SC, USA

## Background

Immunotherapy has recently shown promising clinical activity in multiple tumor types but standard *in vitro* immuno-oncology models for preclinical and clinical predictivity are lacking. Macrophages have been shown to have both tumor promoting and tumor preventing properties dependent upon their differentiation state with M2 macrophages thought to promote tumor growth via enhanced angiogenesis, metastasis and immune evasion. 3D tissue models have been shown to better represent the growth and function of breast tumors and the incorporation of other cell types, such as fibroblasts and adipocytes, and perfusion flow has have been shown by us and others to produce more relevant drug responses. To continue to improve the relevancy of our 3D cancer models we have incorporated macrophages into our current 3D perfusion tri-culture breast tumor model to form a tetra-culture model consisting of cancer cells, fibroblasts, adipocytes, and macrophages.

## Materials and methods

Cancer cell lines, including MCF7, were incorporated into hydrogel/scaffolding combinations along with human mammary fibroblasts and cultured with separate 3D tissues containing either adipocytes or peripheral blood mononuclear cells (PBMC) or differentiated M1, M2 macrophages. These tetra-cultures were grown under perfusion flow for 7 – 21 days and assayed with both destructive and non-destructive methods for survival (Live/Dead), proliferation (PrestoBlue, PicoGreen), morphology (multiphoton microscopy, H&E), marker expression (IHC, RT-PCR), secreted factors (Luminex), motility/migration, and macrophage differentiation states. Additional tests examined drug response changes in the presence of macrophages. Under IRB approved protocols, patient specific tumor cells and immune cells are being incorporated into the 3D tetra-culture models.

## Results

The 3D tumor microenvironment impacted the macrophages by affecting differentiation and survival, and the macrophages affected the tumor cells differently dependent upon whether they were M1 or M2. Initial work has indicated a reduction in tumor cell viability in the presence of M1 macrophages and the reverse in the presence of M2 macrophages. Additionally, morphological changes are evident dependent upon the macrophage presence. Ongoing studies using patient-specific tumor cells and macrophages seek to assess clinically relevant drug response.

## Conclusions

With this 3D tetra-culture model system, we have investigated macrophage differentiation, changes in tumor morphology and survival, and drug response. The use of readily available sources of differentiated macrophages in the 3D co-culture model may assist with preclinical biomarker and novel immuno-oncology drug development, while the matched tumor-macrophage models may serve as personalized ex vivo cell-based immuno-oncology assays used to guide clinical decision making.

